# The identification of a two-gene prognostic model based on cisplatin resistance-related ceRNA network in small cell lung cancer

**DOI:** 10.1186/s12920-023-01536-5

**Published:** 2023-05-15

**Authors:** Yani Zhang, Qizhi Zhu, Jian Qi, Meng Fu, Ao Xu, Wei Wang, Hongzhi Wang, Jinfu Nie, Bo Hong

**Affiliations:** 1 Anhui Province Key Laboratory of Medical Physics and Technology, Institute of Health and Medical Technology, Hefei Institutes of Physical Science, Chinese Academy of Sciences, Hefei, Anhui People’s Republic of China; 2 University of Science and Technology of China, Hefei, Anhui People’s Republic of China; 3 Hefei Cancer Hospital, Chinese Academy of Sciences, Hefei, Anhui People’s Republic of China; 4 Department of Pathology, Division of Life Sciences and Medicine, The First Affiliated Hospital of USTC, University of Science and Technology of China, Hefei, Anhui People’s Republic of China; 5 Division of Life Sciences and Medicine, Intelligent Pathology Institute, University of Science and Technology of China, Hefei, Anhui People’s Republic of China

**Keywords:** Small cell lung cancer, Cisplatin, lncRNA, ceRNA, Prognostic model

## Abstract

**Background:**

Small cell lung cancer (SCLC) is a very malignant tumor with rapid growth and early metastasis. Platinum-based chemo-resistance is the major issue for SCLC treatment failure. Identifying a new prognostic model will help to make an accurate treatment decision for SCLC patients.

**Methods:**

Using the genomics of drug sensitivity in cancer (GDSC) database, we identified cisplatin resistance-related lncRNAs in SCLC cells. Based on the competing endogenous RNA (ceRNA) network, we identified the mRNAs correlated with the lncRNAs. Using Cox and LASSO regression analysis, a prognostic model was established. The survival prediction accuracy was evaluated by receiver operating characteristic (ROC) curve and Kaplan–Meier analysis. GSEA, GO, KEGG and CIBERSORT tools were used for functional enrichment and immune cells infiltration analysis.

**Results:**

We first screened out 10 differentially expressed lncRNAs between cisplatin resistant and sensitive SCLC cells from GDSC database. Based on ceRNA network, 31 mRNAs were identified with a correlation with the 10 lncRNAs. Furthermore, two genes (LIMK2 and PI4K2B) were identified by Cox and LASSO regression analysis to construct a prognostic model. Kaplan–Meier analysis indicated that the high-risk group had a poor overall survival compared with the low-risk group. The predicted area under the ROC curve (AUC) was 0.853 in the training set, and the AUC was 0.671 in the validation set. In the meanwhile, the low expression of LIMK2 or the high expression of PI4K2B in SCLC tumors was also significantly associated with poor overall survival in both training and validation sets. Functional enrichment analysis showed that the low-risk group was enriched in the apoptosis pathway and high immune infiltration of T cells. Finally, an apoptosis-related gene Cathepsin D (CTSD) was identified to be up-regulated in the low-risk group, and its higher expression correlated with better overall survival in SCLC.

**Conclusion:**

We established a prognostic model and potential biomarkers (LIMK2, PI4K2B and CTSD), which could help to improve the risk stratification of SCLC patients.

**Supplementary Information:**

The online version contains supplementary material available at 10.1186/s12920-023-01536-5.

## Introduction

Lung cancer is a growing global problem and the most common cause of cancer-related death [1, 2]. Lung cancer is divided into small cell lung cancer (SCLC) and non-small cell lung cancer (NSCLC). NSCLC accounts for 85% of lung cancers as the most common type of lung cancer [3]. SCLC accounts for only 15% of lung cancer cases, but it is a highly metastatic and recalcitrant neuroendocrine carcinoma [4]. Rapid growth and early metastasis lead to high mortality in SCLC. As a highly aggressive disease, ~ 70% of SCLC cases have been disseminated in the initial clinical presentation, and surgery is only appropriate for a small number of patients with localized disease [5]. The standard first-line treatment for SCLC is platinum-based chemotherapy [6]. Although platinum-based chemo-therapy in SCLC patients with an initial remission rate is as high as 78%, most of the patients recur after 6 months. The median overall survival (OS) of SCLC is only about 10 months [7]. Due to the primary resistance to chemotherapy in a subset of SCLC patients and the acquired resistance to chemotherapy after treatment in almost all SCLC patients, it is desirable to be able to identify SCLC patients with chemo-resistant response. Once these SCLC patients are identified with chemo-resistance and poor survival, immune therapy may be added to improve their survival. Therefore, identifying new biomarkers for chemo-therapy and constructing a prognostic model can help to make an accurate treatment decision for SCLC patients.

Discovering new diagnostic and prognostic biomarkers can be useful to improve risk prediction and therapeutic strategies for cancer patients. The recent study by Giannos et al. identified ten prognostic biomarkers by analyzing TGF-β induced EMT-related genes in NSCLC. Most of these biomarkers were involved in protein ubiquitination, indicating that deregulation of ubiquitination may contribute to the EMT-associated NSCLC progression [8]. In a bioinformatics study of cervical cancer, PCNA gene has been identified as a potential biomarker of cervical intraepithelial neoplasia progression [9]. In esophageal cancer, Feng et al. identified 152 differentially expressed genes between tumors and normal tissues, and construct a 7-gene prognosis model [10].

Long non-coding RNAs (lncRNAs), the non-coding RNAs with a length of more than 200 bp and without protein-coding potential, are tightly linked to regulating cisplatin resistance in various cancers [11]. Many studies have shown that lncRNAs regulate chemo-resistance and cancer progression through sponging miRNAs, which relieves the inhibitory effect of miRNAs on mRNA targets [12]. In SCLC, Sun et al. have reported that lncRNA HOTTIP sponged miR-216a that targeted BCL-2 to increase the expression of BCL-2 and chemoresistance [13]. Zeng et al. have indicated that Linc00173 upregulated Etk by sponging miRNA-218 and caused chemoresistance and poor survival in SCLC [14]. Sun et al. have demonstrated that lncRNA MEG3 sponged miR-15a-5p to mediate CCNE1 expression in cancer-associated fibroblast derived exosomes to promote chemoresistance and cancer progression in SCLC [15]. Therefore, lncRNAs have been demonstrated to play a key role in SCLC chemoresistance by functioning as a competing endogenous RNA (ceRNA).

In the study, through developing a cisplatin resistance-related ceRNA network, we constructed a SCLC prognostic model based on the expression of two mRNAs, LIM domain kinase 2 (LIMK2) and Phosphatidylinositol 4-kinase IIβ (PI4K2B). This model and the two genes (LIMK2 and PI4K2B) have the potential to predict patients’ survival and chemo-resistance in SCLC.

## Methods

### Data acquisition

IC50 values and RNAs expression data of SCLC cell lines were collected from genomics of drug sensitivity in cancer (GDSC, version 8.3, updated in June 2020). The gene expression data based on RNA-seq and clinical information from 77 SCLC patients was obtained from George et al. [16]. The gene expression data and clinical information of 48 SCLC patients from GSE60052 [17] were downloaded from the Gene Expression Omnibus database (GEO). The gene expression data of 43 normal tissues and 15 SCLC tumors from GSE40275 [18] was obtained from GEO.

### The building of cisplatin resistance-related ceRNA network

To build the cisplatin resistance-related ceRNA network, we first identified cisplatin resistance-related lncRNAs. In the GDSC database, with logFC > 0.1 and *P* < 0.05 thresholds, differential expressed lncRNAs were identified between cisplatin-sensitive and cisplatin-resistant group. Subsequently, the miRNAs targeted by lncRNAs were identified in the miRDB database with a target score greater than 80. The mRNAs targeted by miRNAs were identified in miRWalk and NPInterv4 databases, and then the mRNAs obtained by the two databases were intersected. Finally, the correlation of lncRNA-mRNA pairs were examined by Pearson correlation analysis with an absolute value of correlation coefficient > 0.4 and *P* < 0.05.

### Construction of the prognostic model

Using 31 mRNAs identified by ceRNA network, univariate Cox regression analysis was performed to determine the correlation between mRNA expression level and OS in SCLC. The mRNAs with *P* < 0.05 were selected as prognostic genes. LASSO regression analysis was then used to further screen mRNAs and eliminate collinearity between mRNAs. Finally, multivariate Cox regression analysis was used to build a model to obtain the regression coefficient of each mRNA. The risk score was calculated according to the formula:

$${\text{risk score}} = \sum\limits_{i = 1}^{n} {{\text{Coef}}i*xi}$$

“i” is the number of genes, “Coef” is the regression coefficient of this gene, and “x” is the expression value of this gene. The specific risk score = − 1.07*LIMK2 + 0.42*PI4K2B.

Based on the risk scores of patients, the “SurvMiner” package in R was used to calculate the optimal cut-off point and then patients were divided into high-risk and low-risk groups. Kaplan–Meier (KM) survival analysis was performed to assess differences in OS between high-risk and low-risk patients. The “survivalROC” package was used to build the receiver operating characteristic curve (ROC) and calculate the area under ROC curve (AUC). Risk assessment scatter plot showed survival status of patients in high-risk and low-risk groups.

### Functional enrichment analysis

Functional enrichment analysis was performed using R packages including “clusterProfiler”, “org.hs.eg.db”, “GOplot”, “ReactomePA” and “enrichplot”. The George dataset [16] and GSE60052 dataset [17] were analyzed by Gene Set Enrichment Analysis (GSEA) to reveal the biological processes between the high-risk and low-risk group. Subsequently, Gene Ontology (GO) and Kyoto Encyclopedia of Genes and Genomes (KEGG) enrichment analysis [19] were performed on the differentially expressed genes between the high-risk and low-risk group. The pathways with *P* < 0.05 and false discovery rate (q value) < 0.05 were considered to be significant.

### Immune cells infiltration analysis

We use the R package “CIBERSORT” to calculate the different expression levels of immune cells between the high-risk group and low-risk group, and analyze the correlation between the risk score and the level of immune infiltration.

### Immunohistochemical (IHC) staining

The IHC staining was used to examine the expression levels of LIMK2 and PI4K2B in SCLC tumors and normal tissues. Paraffin-embedded sections were incubated in the oven for 3 h. Subsequently, the sections were dewaxed in xylene and ethanol (100%, 95%, 85% and 75%, each for 5 min), and placed in citric acid buffer for antigen repair. After blocked with peroxide blocking solution for 30 min, slides were incubated overnight in a wet chamber at 4 °C with primary antibodies in a concentration of 1:100 (anti-LIMK2, 12350-1-AP, Proteintech, Wuhan, China), and 1:100 (anti-PI4K2B, 15074-1-AP, Proteintech, Wuhan, China). Then, the slides were incubated for 30 min using a secondary antibody (KIT-5020, MaxVision-HRP mouse/rabbit, Maxim, Fuzhou, China). DAB kit (ZLI-9018, Zhongshan Golden Bridge, Beijing, China) was used to stain the sections, and then the slides were counterstained with hematoxylin, dehydrated, and mounted.

### Statistic analysis

All analyses were performed with R version 4.0.2 and corresponding packages. Wilcoxon test and t test were used to compare the difference. *P* < 0.05 was considered to be significant.

## Result

### The establishment of a prognostic model based on the cisplatin resistance-related ceRNA network in SCLC

**Fig. 1:**
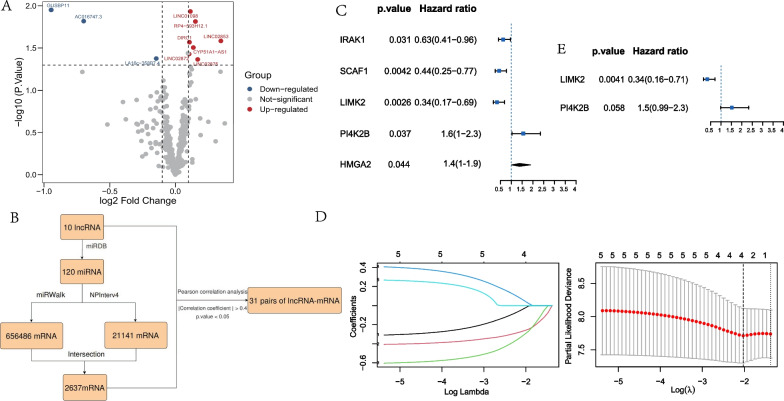
The establishment of a two-gene prognostic model based on cisplatin resistance-related ceRNA network in SCLC. **A** Volcanic map shows differential expression of lncRNAs in cisplatin-resistant and cisplatin-sensitive SCLC cell lines. The red dots represent significantly up-regulated lncRNAs in cisplatin-sensitive cell lines, the blue dots represent significantly down-regulated lncRNAs in cisplatin-sensitive cell lines, and the gray dots represent lncRNAs with no difference (logFC > 0.1 and *P* < 0.05). **B** The flowchart describes the construction of ceRNA network. 31 pairs of lncRNA-mRNA with significant correlation were identified in the network. **C** Univariate Cox regression analysis shows the 5 of 31 cisplatin resistance-related mRNAs associated with OS in George dataset. **D** LASSO regression analysis screened out 4 mRNAs (IRAK1, SCAF1, LIMK2 and PI4K2B). **E** Multivariate Cox regression analysis indentified 2 mRNAs (LIMK2 and PI4K2B) as independent prognostic factors in George dataset

In order to obtain the lncRNA signature related to cisplatin resistance in SCLC, we collected the expression data of lncRNAs in SCLC cell lines from the GDSC database. 53 SCLC cell lines were divided into two groups, 13 cisplatin-sensitive cell lines (cisplatin IC50 < 10) and 40 cisplatin-resistant cell lines (cisplatin IC50 > 10). By comparing the two groups at the thresholds of logFC > 0.1 and *P* < 0.05, 10 differential expressed lncRNAs were identified, including 3 down-regulated lncRNAs (GUSBP11, AC016747 and LA16c-358B7), and 7 up-regulated lncRNAs (RP4-593H12, DIRC1, LINC02872, LINC02875, LINC01098, CYP51A1-AS1 and LINC02853) in cisplatin-sensitive cell lines (Fig. 1A).

Then, we identified 120 miRNAs targeted by the 10 lncRNAs using the miRDB database. Subsequently, mRNAs targeted by the 120 miRNAs were searched in miRWalk and NPInterv4 databases respectively, and 2637 common mRNAs were obtained in both databases (Fig. 1B and Additional file 1: Table S1). Furthermore, Pearson correlation analysis was performed to examine the correlation of lncRNAs and mRNAs expression. Finally, 31 pairs lncRNA-mRNAs were identified with the absolute value of correlation coefficient > 0.4 and *P* < 0.05 (Additional file 2: Table S2).

To establish a prognostic risk model, 31 identified mRNAs were analyzed by univariate Cox regression, LASSO regression and multivariate Cox regression using the SCLC patients’ dataset by George et al. [16]. In univariate Cox regression analysis, five mRNAs (IRAK1, SCAF1, LIMK2, PI4K2B and HMGA2) were significantly associated with OS (Fig. 1C). LASSO regression analysis further screened out 4 mRNAs, including IRAK1, SCAF1, LIMK2 and PI4K2B (Fig. 1D). Then, we performed multivariate Cox regression analysis on the 4 mRNAs and identified the two mRNAs (LIMK2 and PI4K2B), which were independent prognostic factors in SCLC (Fig. 1E). Finally, we used the coefficients of multivariate Cox regression analysis to construct a SCLC prognostic model as the following formula: Risk Score = − 1.07*Exp LIMK2 + 0.42*Exp PI4K2B.

### The validation of the prognostic model in SCLC

**Fig. 2:**
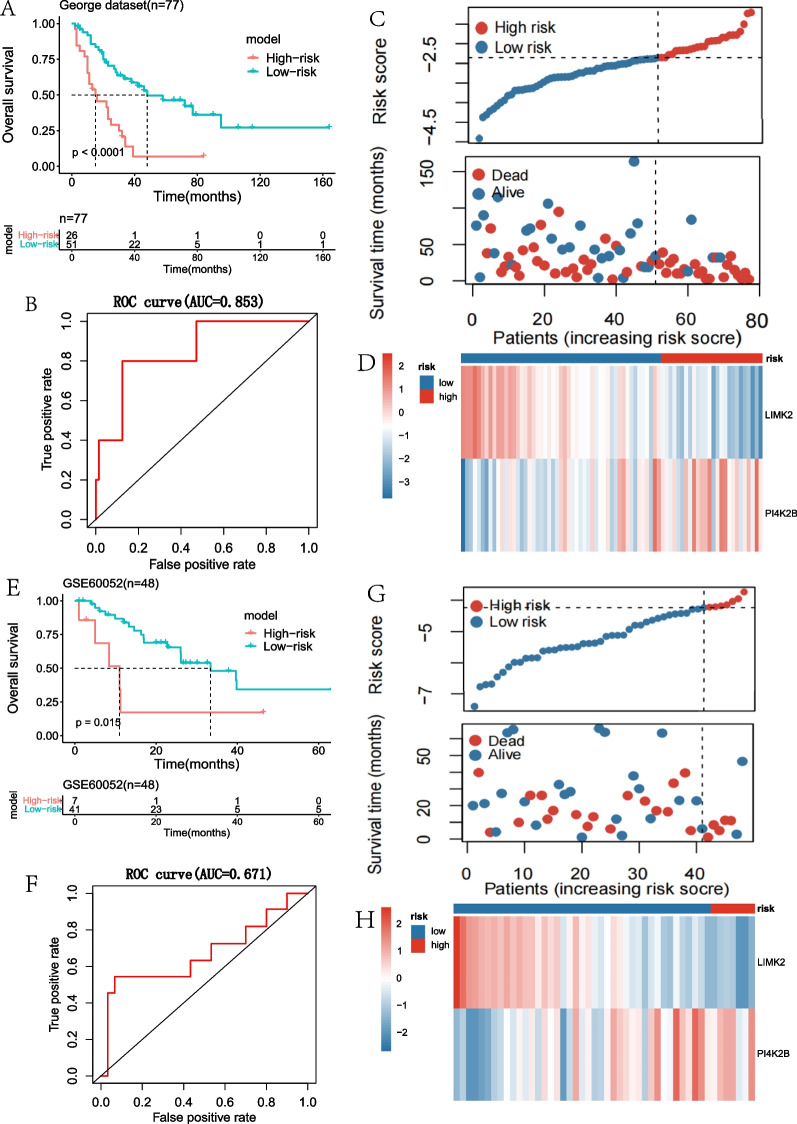
Performance evaluation of the two-gene prognostic model in SCLC. **A** KM survival curve analysis of SCLC patients in George dataset. SCLC patients were divided into low-risk (n = 51) and high-risk (n = 26) groups based on the optimal cut-off point. **B** ROC curve shows AUC in George dataset (n = 77). **C** In George dataset, the risk scores and survival status distribution of SCLC patients. **D** In George dataset, heat map shows the expression of LIMK2 and PI4K2B in SCLC. **E** KM survival curve analysis of SCLC patients in GSE60052 dataset. SCLC patients were divided into low-risk (n = 41) and high-risk (n = 7) groups based on the optimal cut-off point. **F** ROC curve shows AUC in GSE60052 dataset (n = 48). **G** In GSE60052 dataset, the risk scores and survival status distribution of SCLC patients. **H** In GSE60052 dataset, heat map shows the expression of LIMK2 and PI4K2B in SCLC

KM survival analysis and ROC curve analysis were used to evaluate the prognostic value of the model in SCLC. We calculated the risk score of each patient in the George dataset [16] and used the “survminer” package to determine the optimal cut-off score (− 2.36), which divided the patients into a high-risk group (n = 26) and a low-risk group (n = 51). The KM survival analysis showed that the OS in the high-risk group was significantly shorter than that in the low-risk group (Fig. 2A). We then evaluated the predictive power and accuracy of the prognostic risk model according to ROC curve analysis, and the predicted AUC was 0.853 (Fig. 2B). The risk score and survival status distribution of each patient were shown in Fig. 2C, indicating that the patients with higher risk scores were more likely to have a poorer prognosis compared to patients with the lower risk scores. The expression heatmap showed that LIMK2 expression was higher in the low-risk group, while PI4K2B expression was lower in the low-risk group (Fig. 2D).

To further validate the prognostic value of the model in SCLC patients, we analyzed an independent dataset (GSE60052) with 48 SCLC patients. Similarly, the patients from the dataset were divided into the high-risk group (n = 7) and a low-risk group (n = 41) by the optimal cut-off of risk scores (− 4.23). The results of KM survival analysis showed that the OS of high-risk group was significantly shorter than that of the low-risk group (Fig. 2E). The predicted AUC value was 0.671 (Fig. 2F). The risk score and survival status distribution of each patient were shown in Fig. 2G. Similar to the George dataset [16], LIMK2 expression was higher and PI4K2B expression was lower in the low-risk group (Fig. 2H). Thus, these data indicate that the model based on the expressions of LIMK2 and PI4K2B performs a good prognostic value in SCLC.

### The assessment of the prognostic value of the single LIMK2 or PI4K2B gene in SCLC

**Fig. 3:**
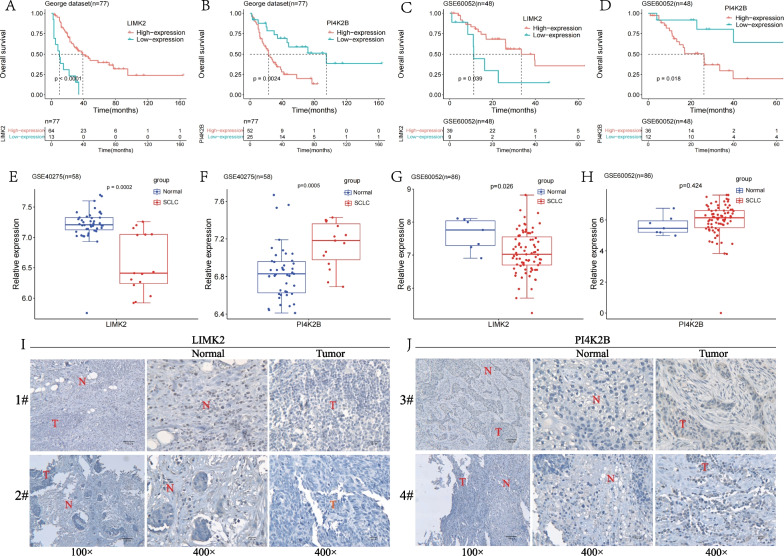
Prognostic value of single LIMK2 or PI4K2B gene in SCLC. **A** KM survival analysis of LIMK2 in George dataset (n = 77). SCLC patients were divided into low-expression (n = 13) and high-expression (n = 64) groups based on the optimal cut-off point. **B** KM survival analysis of PI4K2B in George dataset (n = 77). SCLC patients were divided into low-expression (n = 25) and high-expression (n = 52) groups based on the optimal cut-off point. **C** KM survival analysis of LIMK2 in GSE60052 dataset (n = 48). SCLC patients were divided into low-expression (n = 9) and high-expression (n = 39) groups based on the optimal cut-off point. **D** KM survival analysis of PI4K2B in GSE60052 dataset (n = 48). SCLC patients were divided into low-expression (n = 12) and high-expression (n = 36) groups based on the optimal cut-off point. **E** In GSE40275 dataset, boxplot of LIMK2 mRNA expression in SCLC tumors and normal tissues. **F** In GSE40275 dataset, boxplot of PI4K2B mRNA expression in SCLC tumors and normal tissues. **G** In GSE60052 dataset, boxplot of LIMK2 mRNA expression in SCLC tumors and normal tissues. **H** In GSE60052 dataset, boxplot of PI4K2B mRNA expression in SCLC tumors and normal tissues. **I** IHC detection of LIMK2 expression in two SCLC clinical specimens (T: Tumor, N: Normal tissue). **J** IHC detection of PI4K2B expression in two SCLC clinical specimens (T: Tumor, N: Normal tissue)

Furthermore, we evaluated the prognostic value of the single LIMK2 or PI4K2B gene in SCLC. KM analysis showed that low expression of LIMK2 was significantly associated with poor survival in the both George [16] (Fig. 3A) and GSE60052 data sets (Fig. 3C). High expression of PI4K2B was significantly associated with poor survival in the both George [16] (Fig. 3B) and GSE60052 data sets (Fig. 3D). These results were consistent with the hazard ratio (HR) of Cox regression analysis on the two genes (LIMK2 and PI4K2B).

Next, we evaluated the expression of LIMK2 and PI4K2B in SCLC tumor and normal tissues by using GSE40275 and GSE60052 datasets. The data indicated that mRNA level of LIMK2 was significantly higher in normal tissues in both datasets (Fig. 3E, G). The mRNA level of PI4K2B was significantly higher in SCLC tumors in GSE40275 dataset (Fig. 3F). In GSE60052 dataset, mRNA level of PI4K2B was higher in SCLC tumors, but the difference did not reach statistic significance (Fig. 3H). Furthermore, the expression of LIMK2 and PI4K2B was verified in our collected SCLC specimens by IHC. The IHC data showed that the expression of LIMK2 was down-regulated in SCLC tumors, compared with normal tissues. LINK2 was primarily expressed in the nucleus of normal cells (Fig. 3I). However, the expression of PI4K2B was up-regulated in SCLC tumors, compared with normal tissues. PI4K2B was primarily expressed in the cytoplasm of SCLC cells (Fig. 3J). Therefore, the mRNA levels of LIMK2 and PI4K2B in SCLC tumors and normal tissues were consistent with the IHC data.

### Functional enrichment analysis of the model

**Fig. 4:**
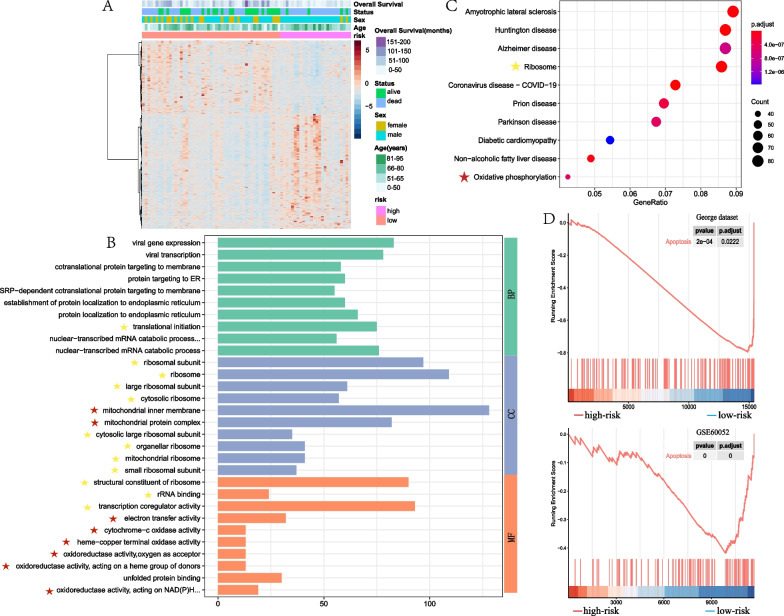
Functional enrichment analysis of the prognostic model. **A** Heat map of differentially expressed genes between low-risk group and high-risk group in George dataset. **B** Functional enrichment analysis of GO terms for the differentially expressed genes between low-risk group and high-risk group in George dataset. Yellow stars indicate the terms associated with ribosomes, and red stars indicate the terms associated with mitochondria. **C** Functional enrichment analysis of KEGG for the differentially expressed genes between low-risk group and high-risk group in George dataset. Yellow stars indicate the terms associated with ribosomes, and red stars indicate the terms associated with mitochondria. **D** GSEA enrichment analysis of both George dataset and GSE60052 dataset shows that the apoptotic pathway is enriched in the low risk group

To elucidate the biological functions and potential pathways related to the risk model, we conducted GO and KEGG enrichment analyses based on differentially expressed genes between the high-risk and low-risk group. In the George dataset [16], according to the threshold of logFC > 1 and *P* < 0.05, 2266 differentially expressed genes were identified between the high-risk and low-risk group (Fig. 4A). The GO term analysis of these differentially expressed genes revealed the functional enrichment of mitochondrial and ribosome activities, including electron transfer activity, cytochrome C oxidase activity and translational initiation et al. (Fig. 4B). The KEGG analysis of these genes also enriched the terms of mitochondrial oxidative phosphorylation and ribosome terms (Fig. 4C).

Furthermore, we employed GSEA to analyze the biological process and signaling pathway enriched in the high-risk and low-risk groups. All genes were sorted in ascending order according to the logFC between the two groups, and then they were subjected to GSEA enrichment analysis. In both George [16] and GSE60052 dataset, the apoptosis pathway was enriched in the low-risk group (Fig. 4D). The mitochondrial is an important organelle to regulate apoptosis. Therefore, the results of functional enrichment analyses suggest that the prognostic model may be related to mitochondrial-regulated cellular apoptosis.

### Cathepsin D (CTSD) is an important apoptosis-related gene enriched in the model

**Fig. 5:**
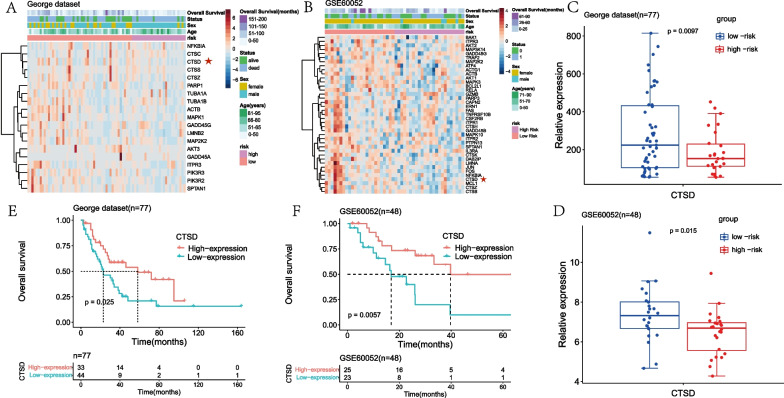
CTSD is identified as an important apoptotic regulator in both George dataset and GSE60052 dataset. **A** In George dataset, heat map of 19 differential expressed core genes of apoptotic pathway between the low-risk and high-risk group. **B** In GSE60052 dataset, heat map of 39 differential expressed core genes of apoptotic pathway between the low-risk and high-risk group. **C** In George dataset, boxplot of CTSD expression in the low-risk and high-risk groups **D** In GSE60052 dataset, boxplot of CTSD expression in the low-risk and high-risk groups. **E** KM survival analysis of CTSD in George dataset. SCLC patients were divided into low-expression (n = 44) and high-expression (n = 33) groups based on the optimal cut-off point. **F** KM survival analysis of CTSD in GSE60052 dataset. SCLC patients were divided into low-expression (n = 23) and high-expression (n = 25) groups based on the optimal cut-off point

In order to explore the key proteins of the apoptosis pathway enriched in the model, we analyzed the core genes in the apoptosis pathway between the high-risk and low-risk group. According to the threshold of LogFC > 0.1 and *P* < 0.05, 19 key apoptosis-related genes were identified in the George dataset [16], and 39 key apoptosis-related genes were identified in the GSE60052 dataset (Fig. 5A, B). Among these genes, CTSD was down-regulated in high-risk group in both datasets (Fig. 5C, D). Moreover, we evaluated the prognostic value of CTSD in SCLC. KM analysis indicated that the low expression of CTSD was associated with poor OS in both George [16] and GSE60052 datasets (Fig. 5E, F). Therefore, these results suggest that CTSD could be an important apoptotic modulator enriched in the model.

### The immune infiltrating analysis of the risk groups in SCLC

**Fig. 6:**
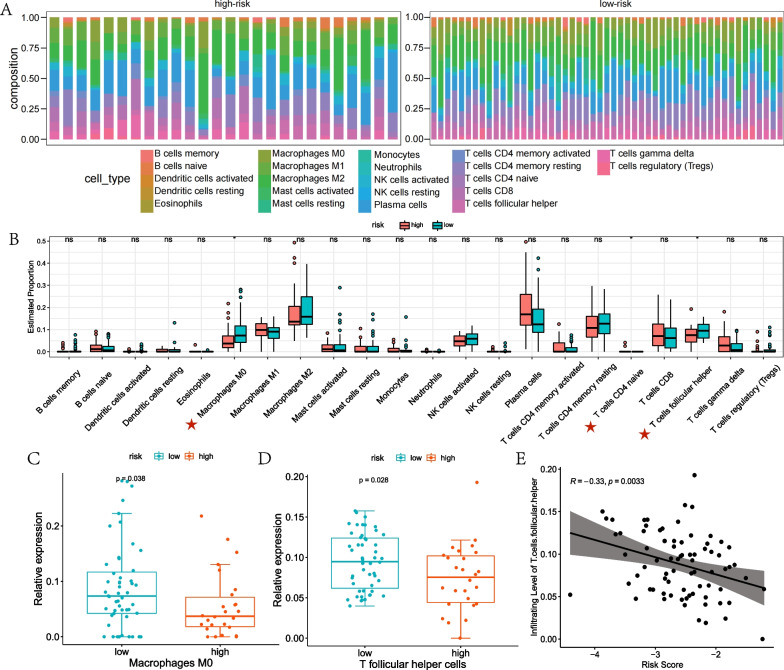
The immune infiltrating analysis of the risk groups in SCLC. **A** In George dataset, landscape of different immune cell subtypes for each sample (n = 77). **B** In George dataset, the differences in the level of 22 immune cell infiltration between high-risk group (n = 26) and low-risk group (n = 51). **C** Macrophages M0 cell infiltration with significantly different levels between the high risk group and low risk group. **D** T follicular helper cells infiltration with significantly different levels between the high risk group and low risk group. **E** Pearson correlation analysis of T follicular helper cells infiltration and risk score of each SCLC sample

The infiltrating levels of 22 immune cells in the risk group of SCLC were explored using the CIBERSORT algorithm. We constructed the landscapes of different immune cell subtypes in each sample (Fig. 6A). The data demonstrated that the levels of macrophages M0 and T follicular helper cells were significantly higher in the low-risk group compared with the high-risk group (Fig. 6B–D). Moreover, the correlation analysis between the risk scores and 22 immune infiltrating cells demonstrated that the risk score was significantly related to the infiltration level of T follicular helper cells. With the risk score increased, the infiltrating level of T follicular helper cells decreased (Fig. 6E). The data suggest that the high risk group of the model is related to low immune cells infiltration.

### The ceRNA network of the SCLC prognostic model

**Fig. 7:**
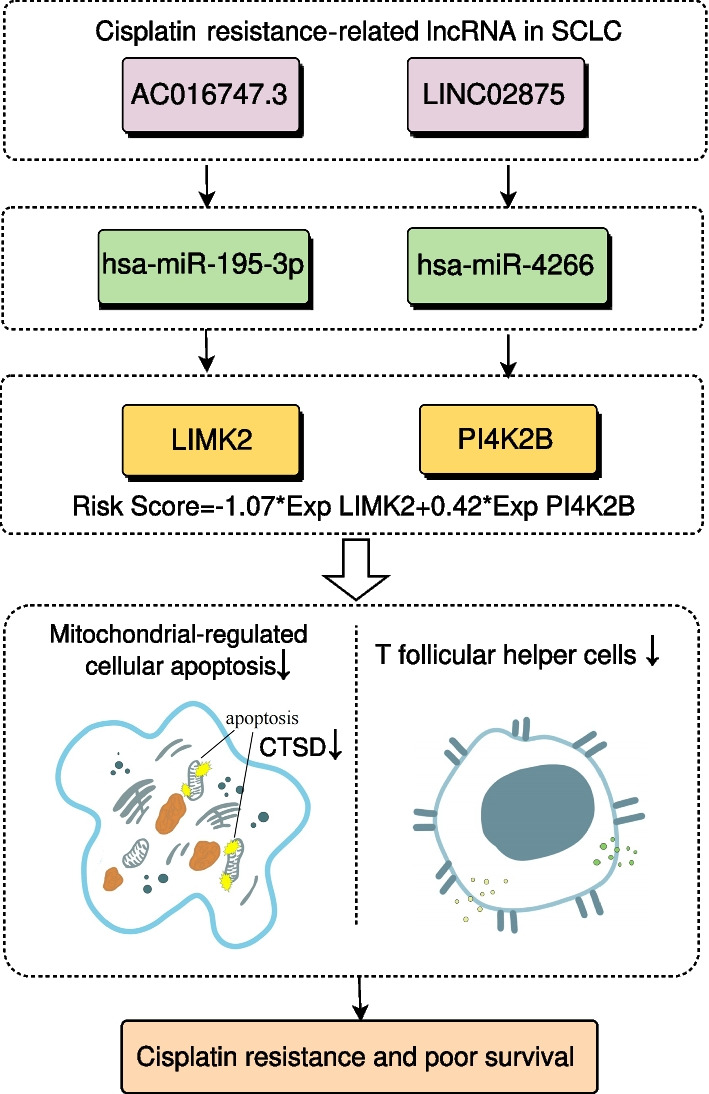
The cisplatin resistance-related ceRNA network and possible functional mechanism. The cisplatin resistance-related ceRNA network AC016747.3/hsa-miR-195-3p/LIMK2 and LINC02875/hsa-miR-4266/PI4K2B could affect mitochondrial-regulated cellular apoptosis and T cell immune infiltration, thereby leading to cisplatin resistance and poor survival in SCLC

Taken together, these results identified cisplatin resistance-related ceRNA network (AC016747.3/hsa-miR-195-3p/LIMK2 and LINC02875/hsa-miR-4266/PI4K2B) in SCLC. In the meanwhile, based on the ceRNA network, we established a two-gene prognostic model, which had a good ability to predict the survival of SCLC patients. Furthermore, the identified ceRNA network may affect cisplatin resistance and patients’ survival through mitochondrial-regulated apoptosis and immune infiltration (Fig. 7).

## Discussion

In this study, we first identified 10 cisplatin resistance-related lncRNAs in SCLC cell lines. Through the ceRNA network and correlation analysis, we found 31 mRNAs correlated with the 10 lncRNAs. Then, using Cox regression analysis, we established a prognostic model based on the expression of LIMK2 and PI4K2B genes in SCLC. Furthermore, functional enrichment analysis found that the risk groups in the model could be related to mitochondrial-regulated cellular apoptosis pathway and T cells infiltration.

In our model, the higher expression of LIMK2 gene correlated with better survival in SCLC. As previously reported, LIMK2 was a serine/threonine protein kinase that positively associated with OS in lung squamous cell carcinoma (LUSC), which was consistent with our findings. The previous study indicated that LIMK2 expression negatively correlated with immune checkpoints and immune cell infiltration in LUSC, while our study showed that the low risk group (high LIMK2 expression) exhibited high immune cell infiltration. The study also found a DHRS4-AS1/miR-423-5p/LIMK2 ceRNA axis in LUSC, while our study found an AC016747.3/hsa-miR-195-3p/LIMK2 ceRNA axis in SCLC [20, 21]. Our model suggests that LIMK2 acts as a tumor suppressor gene. Previous studies have demonstrated that low expression of LIMK2 in colorectal cancer enhanced the accumulation of β-catenin in the nucleus to activate Wnt signaling pathway and promote tumor progression, while high expression of LIMK2 inhibited the proliferation and migration of tumor cells [22]. However, some studies also reported that LIMK2 acted as an oncogene. For instance, LIMK2 downregulated NKX3.1 to increase the oncogenicity of castration-resistant prostate cancer [23], and overexpressed LIMK2 exhibited as a facilitator of triple-negative breast cancer metastasis by regulating SRPK1 [24].

In our model, the lower expression of PI4K2B gene correlated with better survival in SCLC, which suggests that PI4K2B may function as an oncogene in SCLC. Mazzocca et al. have indicated that PI4K2B and CD81 could synergistically inhibit the migration of liver cancer cells, and low expression of PI4K2B increased the migration of liver cancer cells [25]. Alli-Balogun et al. also demonstrated the effect of PI4K2B on the movement of Hela cells, and they found that depletion of PI4K2B induced the formation of invadopodia containing matrix metalloproteinase, to increase cellular invasion [26]. On the contrary, these previous studies have indicated that PI4K2B may function as a tumor suppressor to inhibit tumor invasion.

In order to explore the mechanistic role of the model in SCLC, GO and KEGG enrichment analysis indicated that the differentially expressed genes between high-risk group and low-risk group were enriched in the mitochondrial function. GSEA analysis showed that the apoptotic pathway was enriched in the low-risk group. Cisplatin-based chemotherapy works by forming platinum–DNA adducts, which prevent rapidly dividing cells from duplicating their DNA, thereby leading to cellular apoptosis. The inhibition of apoptosis is an important mechanism for cancer cells to evade cisplatin toxicity and lead to drug resistance [27, 28]. The construction of the model is based on cisplatin resistance-related ceRNA network. Consistently, the functional mechanism of the prognostic model could be related to mitochondrial-regulated apoptotic pathway.

In this study, we identified CTSD as a key regulator of apoptosis in SCLC. CTSD is an aspartic endopeptidase [29, 30]. Previous studies have shown that CTSD responds to apoptosis stimulation. CTSD is located upstream of cytochrome C release and caspase-3 activation in chemotherapy-induced apoptosis pathway [31–33]. Secomandi et al. have indicated that the overexpression of CTSD in neuroblastoma reduced the proliferation of tumor cells by down-regulating oncogenic MAPK signaling pathway, and the high expression of CTSD correlated with better prognosis [34]. In our study, the higher expression of CTSD was also associated with a better prognosis of SCLC. Therefore, our study suggests that CTSD plays a critical role in the response to the chemotherapy of SCLC.

The study has also some limitations. Firstly, the prognostic model was built based on the bioinformatics analysis of publicly available datasets, and the results need to be further validated in prospective studies with a large sample size. Secondly, the study only focused on two genes (LIMK2 and PI4K2B) and their relationship with apoptosis pathway and immune infiltration. Other identified genes (such as IRAK1, SCAF1 and HMGA2) and pathways (such as protein translation in ribosome) may be also important in the chemo-resistance and progression of SCLC. Thirdly, the biological function and mechanism of the identified ceRNA network and biomarkers need to be validated using in vitro and in vivo experiments in the future.

## Conclusion

Based on cisplatin resistance-related ceRNA network, our study established a two-gene (LIMK2 and PI4K2B) prognostic model in SCLC. The functional role of the model was related to mitochondrial-regulated apoptosis and immune cell infiltration. Furthermore, we identified an important apoptosis-related protein CTSD, whose expression positively associated with OS in SCLC. Therefore, the two-gene prognostic model and these new identified biomarkers (LIMK2, PI4K2B and CTSD) could provide an accurate treatment decision for SCLC patients.

## Supplementary Information

**Additional file 1. Table S1**: 4465 lncRNAs-miRNA-mRNA axes in the cisplatin resistance-related ceRNA network.

**Additional file 2. Table S2**: 31 pairs lncRNA-mRNAs.

## Data Availability

This study analyzed publicly available datasets. These data can be found here: https://www.cancerrxgene.org and https://www.ncbi.nlm.nih.gov/geo.

## Abbreviations

SCLC: Small cell lung cancer
NSCLC: Non-small cell lung cancer
OS: Overall survival
LncRNA: Long non-coding RNA
ceRNA: Competing endogenous RNA
LIMK2: LIM domain kinase 2
PI4K2B: Phosphatidylinositol 4-kinase IIβ
GDSC: Genomics of drug sensitivity in cancer
GEO: Gene expression omnibus database
KM: Kaplan–Meier
ROC: Receiver operating characteristic
AUC: Areas under the ROC curves
GSEA: Gene set enrichment analysis
GO: Gene ontology
KEGG: Kyoto encyclopedia of genes and genomes
HR: Hazard ratio
CTSD: Cathepsin D
